# *In-Vitro* Differentiation of Mature Dendritic Cells From Human Blood Monocytes

**DOI:** 10.1155/1998/72054

**Published:** 1998

**Authors:** Robert Gieseler, Dirk Heise, Afsaneh Soruri, Peter Schwartz, J. Hinrich Peters

**Affiliations:** 1 Department of Immunology Georg-August University Göttingen D-37075 Germany; 2 lnstitute for Anatomy Georg-August University Göttingen D-37075 Germany; 3 Division of Clinical Endocrinology Department of Medicine University Clinics Hufelandstrasse 55 Essen D-45122 Germany

**Keywords:** Dendritic cells, GM-CSF, interferon %, interleukin-4, macrophages, mucosa

## Abstract

Representing the most potent antigen-presenting cells, dendritic cells (DC) can now be generated from human blood monocytes. We recently presented a novel protocol employing GM-CSF, IL-4, and IFN-γ to differentiate monocyte-derived DC *in vitro*. Here, such cells are characterized in detail. Cells in culture exhibited both dendritic and veiled morphologies, the former being adherent and the latter suspended. Phenotypically, they were CD1a^-/dim^, CD11a^+^, CD11b^++^, CD11c^+^, CD14^dim/-^, CD16^a-/dim^, CD18^+^, CD32^dim/-^, CD33^+^, CD40^+^, CD45R0^+^, CD50^+^, CD54^+^, CD64^-/dim^, CD68^+^, CD71^+^, CD80^dim^, CD86^+/++^, MHC class I^++/+++^ HLA-DR^++/+++^ HLA-DP^+^, and HLA-DQ^+^. The DC stimulated a strong allogeneic T-cell response, and further evidence for their autologous antigen-specific stimulation is discussed. Although resembling a mature CD 11c^+^CD45R0^+^ blood DC subset identified earlier, their differentiation in the presence of the Thl and Th2 cytokines IFN-γ and IL-4 indicates that these DC may conform to mature mucosal DC.


*Developmental Immunology*, 1998, Vol. 6, pp. 25–39
Reprints available directly from the publisher
Photocopying permitted by license only

© 1998 OPA (Overseas Publishers Association)
N.V. Published by license under the
Harwood Academic Publishers imprint,
part of the Gordon and Breach Publishing Group
Printed in Malaysia

### *In-Vitro* Differentiation of Mature Dendritic Cells from Human Blood Monocytes

ROBERT GIESELER[a*], DIRK HEISE[a], AFSANEH SORURI[a], PETER SCHWARTZ[b] and J. HINRICH PETERS[a]

[a]*Department of Immunology*; [b]*Institute for Anatomy*; *Georg-August University, D-37075 Göttingen, Germany*

*(Received 15 August 1996; Accepted 14 April 1997)*

Representing the most potent antigen-presenting cells, dendritic cells (DC) can now be generated from human blood monocytes. We recently presented a novel protocol employing GM-CSF, IL-4, and IFN-$\gamma$ to differentiate monocyte-derived DC *in vitro*. Here, such cells are characterized in detail. Cells in culture exhibited both dendritic and veiled morphologies, the former being adherent and the latter suspended. Phenotypically, they were CD1a$^{-/dim}$, CD11a$^{+}$, CD11b$^{++}$, CD11c$^{+}$, CD14$^{dim/-}$, CD16a$^{-/dim}$, CD18$^{+}$, CD32$^{dim/-}$, CD33$^{+}$, CD40$^{+}$, CD45R0$^{+}$, CD50$^{+}$, CD54$^{+}$, CD64$^{-/dim}$, CD68$^{+}$, CD71$^{+}$, CD80$^{dim}$, CD86$^{+/++}$, MHC class I$^{++/+++}$, HLA-DR$^{++/+++}$, HLA-DP$^{+}$, and HLA-DQ$^{+}$. The DC stimulated a strong allogeneic T-cell response, and further evidence for their autologous antigen-specific stimulation is discussed. Although resembling a mature CD11c$^{+}$CD45R0$^{+}$ blood DC subset identified earlier, their differentiation in the presence of the Th1 and Th2 cytokines IFN-$\gamma$ and IL-4 indicates that these DC may conform to mature mucosal DC.

*Keywords:* Dendritic cells, GM-CSF, interferon $\gamma$, interleukin-4, macrophages, mucosa

### INTRODUCTION

We recently presented cumulative evidence for the myelomonocytic origin of dendritic cells (DC) that belong to the functional entity of professional antigen-presenting cells (APC) (Peters et al., 1996). Partly resolving the controversy on the ontogenetic pathway of DC recruitment, there is now sufficient proof for their differentiation from myeloid precursors. Accordingly, both DC or their migratory counterparts expressing cytoplasmic veils, were generated *in vitro* both from myeloid-lineage-type bone marrow precursors (Gieseler et al., 1991; Reid et al., 1992; Santiago-Schwarz et al., 1992; Inaba et al., 1993; Romani et al., 1994; Chen-Woan et al., 1996) as well as late blood monocytes (Gieseler, 1987; Peters et al., 1987; Kabel et al., 1989; Thomas et al., 1993; Zhou and Tedder, 1996).

Thus, large numbers of DC can now be generated from myelomonocytic progeny. It should be noted, however, that this does not rule out the possible differentiation of DC from nonmyeloid lineages

*Corresponding author. Present address: Robert Gieseler, Division of Clinical Endocrinology, Department of Medicine, University Clinics, Hufelandstrasse 55, D-45122 Essen Germany.
RG and DH contributed equally to this work and should be considered first co-authors.

(Steinman and Cohn, 1973; Steinman, 1991), perhaps even common with T-cell progenitors (Ardavin et al., 1993; O'Neill, 1994). Future studies will help to classify such observations, which may, for the time being, be regarded as hints toward the complex regulation and integration of antigen specificity.

In any case, depending on the nature and composition of environmental stimuli, monocytes may be induced to differentiate into either of the two major classes of professional APC, that is, DC or macrophages (M$\varphi$) (Peters et al., 1991, 1996). Because DC and M$\varphi$ are crucial for directing the course of immunity, the genetic program underlying their developmental dichotomy is to be regarded as a major cornerstone of immune regulation.

Moreover, as to the diverse conditions in the various anatomic sites, local monocyte differentiation should even result in a broad spectrum of phenotypes, rather than only two idealized cell populations. Indeed, this deduction has long been verified empirically, suggesting that the actual tissue shapes its characteristic APC compartment (e.g., Franklin et al., 1986). This is consistent with the finding that, for example, DC obtained from spleen and Peyer's patches profoundly differ in their preferential activation of T-helper 1 (Th1) or Th2 cells (Everson et al., 1996).

Studying the effect of growth factors and cytokines on isolated monocytes thus appears suitable to identify signals controlling the development of discrete myeloid APC types. As to the thought pathway sketched before, such molecules would induce basic DC or M$\varphi$ differentiation as well as fine-tune their tissue-specific maturation. The past years then brought about several culture systems for the generation of monocyte-derived DC (MoDC) in the presence of selected factors (cf. Peters et al., 1996). One of the generally accepted protocols employs a combination of granulocyte-macrophage colony-stimulating factor (GM-CSF) and interleukin-4 (IL-4), leading to CD1a$^{+}$ and CD14$^{-}$ or CD14$^{dim}$ DC that are able to induce mycobacterium- and tetanus-toxin-specific T-cell proliferation, as well as mixed leukocyte responses (Porcelli et al., 1992; Ruppert et al., 1993; Romani et al., 1994; Sallusto and Lanzavecchia, 1994; Steinbach et al., 1995; Kiertscher and Roth, 1996).

We then proceeded to question whether interferon $\gamma$ (IFN-$\gamma$), which upregulates the expression of major histocompatibility complex (MHC) class II molecules, might support the functionality of MoDC. This working hypothesis could be verified when culturing monocytes with GM-CSF, IL-4, and IFN-$\gamma$, which gave rise to DC strongly supporting a mixed leukocyte reaction (Xu et al., 1995).

This paper now presents a phenotypic characterization of freshly isolated monocytes, and the MoDC (including both DC and veiled cells) generated from them by action of GM-CSF, IL-4, and IFN-$\gamma$. Specifically, we focused on the expression of myeloid-type markers, MHC class I and II antigens, selected adhesion molecules, as well as antigens delineating certain subsets and maturity stages of DC *in vivo*. The resultant phenotypic profile now allows to classify the ontogenetic root of such cells, to pinpoint their stage of development and to discuss their possible counterpart *in vivo*. Additional morphological and functional data underscore the mature stage of the MoDC obtained with this culture system.

### RESULTS

### Morphology and Differentiation of MoDC in Culture

Monocytes cultured for 6 days in the presence of GM-CSF, IL-4, and IFN-$\gamma$ exhibited three principal morphologies. The majority of cells (> 60%) weakly adhered to the gas-permeable Teflon surface of the culture flasks. These cells revealed numerous dendritic projections and occasional veillike cytoplasmic structures. Their general morphologic appearance resembled that of Langerhans cells *in situ*.

Starting with the third day of culture, part of such cells began to detach and gave rise to a second population of suspended cells that, 1 to 2 days later, plateaued at ~30% of the total cell number. These nonadherent cells had ovoid, bluntly branched cell bodies exhibiting abundant cytoplasmic veils. Veil structures appeared to mainly bud from such projections (Figure 1), which may correspond to the dendritiform ramifications during the adherent cell stage.

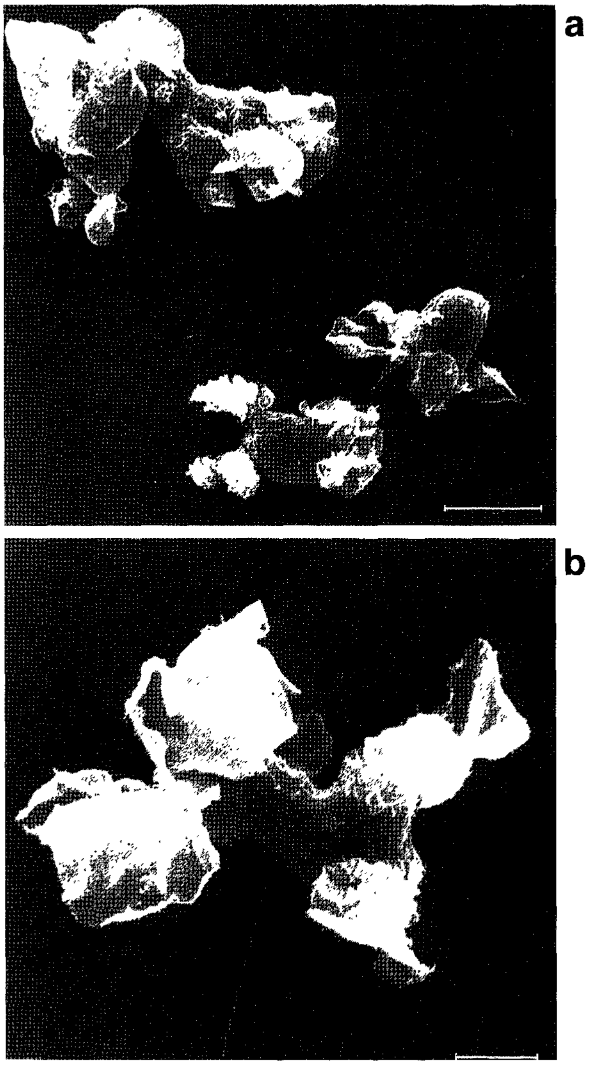

FIGURE 1 Scanning electron micrographs of veiled cells derived from blood monocytes. Veiled cells were collected from supernatants of cells cultured for 6 days in the presence of GM-CSF, IL-4, and IFN-$\gamma$, and processed as indicated. (a) Several cells immobilized on poly-L-lysine exhibit a spectrum of veil structures and cytoplasmic processes. Original magnification × 1500. The bar represents 10 $\mu$m. (b) The typical appearance of a suspended cell in culture conforms to blood dendritic cells (Knight et al., 1986) or veiled cells from afferent lymph (Barfoot et al., 1989) or colonic lamina propria (Pavli et al., 1993). Original magnification × 3000. The bar indicates 5 $\mu$m.

Third, we observed a small proportion of ~5% strongly adherent M$\varphi$. The majority of these cells were stretched in shape with a broad footlike projection on one of the cell poles. M$\varphi$ were larger than dendritic and veiled cells and, unlike these, exhibited abundant perinuclear lysosomes and occasional vacuolization. These M$\varphi$ revealed some very interesting features deserving special consideration and are presented in detail elsewhere (Gieseler et al., 1998).

### The Phenotype of MoDC

Depending on their mean fluorescence intensities ($\Delta$MFI; see Materials and Methods), we here defined the expression of markers as dim ($\Delta$MFI 10-100), + ($\Delta$MFI > 100-1000), ++ ($\Delta$MFI > 1000-5000) or +++ ($\Delta$MFI > 5000).

### Myeloid Antigens

Cultured cells did not proliferate, that is, they did not form clones or incorporate [$^3$H]thymidine (not shown). Hence, DC and veiled cells generated in the presence of cytokines clearly were descendants of monocytes, as shown by their expression of the myeloid lineage marker CD33 (throughout the myeloid life cycle) as well as CD68 (Figure 2). Though often being considered a M$\varphi$-specific marker, CD68 may also be expressed by DC (Beelen et al., 1993). However, CD68 expression on MoDC was lower than that observed on serum-cultured M$\varphi$ (Figures 2B and 2C). Another marker still regarded as a hallmark of M$\varphi$ is CD14, that is, the receptor for complexes of LPS/LPS-binding protein. Yet, *in vivo*, DC in distinct sites, such as mucosal tissue, may well express CD14, as was shown by Graeme-Cook et al. (1993). Therefore, supposing a common origin of DC and M$\varphi$, CD68 and CD14 may be subject to exogenous environmental regulation in either of these populations and their site-specific subsets. Earlier, Thomas et al. (1993) showed that blood DC are CD14$^{dim}$CD33$^{bright}$. Accordingly, in confirming and extending results from Ruppert et al., (1993), Sallusto and Lanzavecchia (1994) as well as Kiertscher and

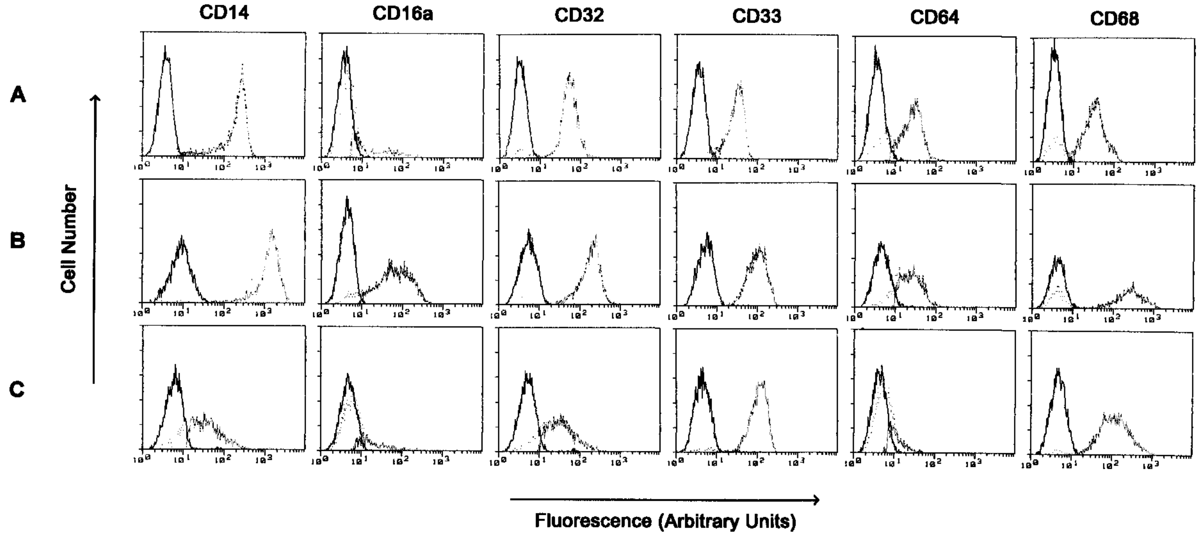

FIGURE 2 Expression of myeloid antigens as determined by flow cytometry. The panel compares antigen expression by monocytes (row A), monocytes cultured for 6 days with serum alone (row B), and DC and veiled cells derived from monocytes by action of GM-CSF/IL-4/IFN-$\gamma$ (row C). The mAb are detailed in Table I. Each graph is representative of four to six experiments carried out. Histograms depict the number of cells (maximal scaling equals to 200 cells) exhibiting various fluorescence intensities ($\Delta$MFI) with either control mAb (bold lines on the left) or tested antigens (faint lines). Percentages of antigen-positive cells in Fig. 6 were calculated from these data using the C30 program.

Roth (1996) obtained with GM-CSF and IL-4, we here show complete downregulation of CD14 on 28.4% of MoDC as well as a CD14$^{dim}$ phenotype of the remaining cells (Figure 2C). Also, the Fc$\gamma$ receptors (FcR) type I, II, and IIIA (CD64, CD32, CD16a) were found downregulated. Very faint expression of CD16 and CD64 (Figure 2C) by 33.9% or 24.6% of cells (Figure 6) cultured with GM-CSF/IL-4/IFN-$\gamma$ was partly due to the small M$\varphi$ subpopulation that coemerged from the starter monocytes (see earlier), and some of the MoDC, but not the veiled cells. An interesting result was the CD32$^{dim}$ expression in 78.4% of cells (Figures 2C and 6), because this conforms to blood DC expressing the maturity marker CD83 (Zhou and Tedder, 1995). The remaining cells were CD32$^{-}$. Thus, according to the preceding definition, the myeloid antigen pattern detected on MoDC generated in the presence of GM-CSF, IL-4, and IFN-$\gamma$ was CD14$^{dim/-}$, CD16a$^{-/dim}$, CD32$^{dim/-}$, CD33$^{+}$, CD64$^{-/dim}$, and CD68$^{+}$. Double statements indicate the expression by different stages of maturity, with the major portion mentioned first and the minor portion in the second position.

### DC and Maturation Antigens

Certain populations of DC or subsets thereof can be discerned by several markers. The most prominent may be CD1a, which is expressed on immature epidermal Langerhans cells, but becomes abrogated when the cells drain to the lymph nodes and mature. Here, 9.0-32.6% of MoDC were CD1a$^{dim}$ (Figures 6 and 3C). We did not follow CD1a expression during the previous days of culture. However, judging from the maturity markers determined in this study, the majority of MoDC cultured for 6 days appear to be in a mature state that, therefore, does not conflict with the loss of CD1a. Two of such markers are the integrin $\alpha_X$-chain CD11c and CD45R0 — a splicing variant of the leukocyte common antigen. CD45R0 is not only expressed by activated or memory T and B cells, but also by mature blood DC (Zhou and Tedder, 1995) as well as DC differentiated from monocytes in the presence of GM-CSF, IL-4, and tumor necrosis factor $\alpha$ (TNF-$\alpha$) (Zhou and Tedder, 1996). In 1994, O'Doherty et al. (1993) showed that a subpopulation of blood DC express both, CD11c and CD45R0, which reasonably is taken as circumstantial evidence

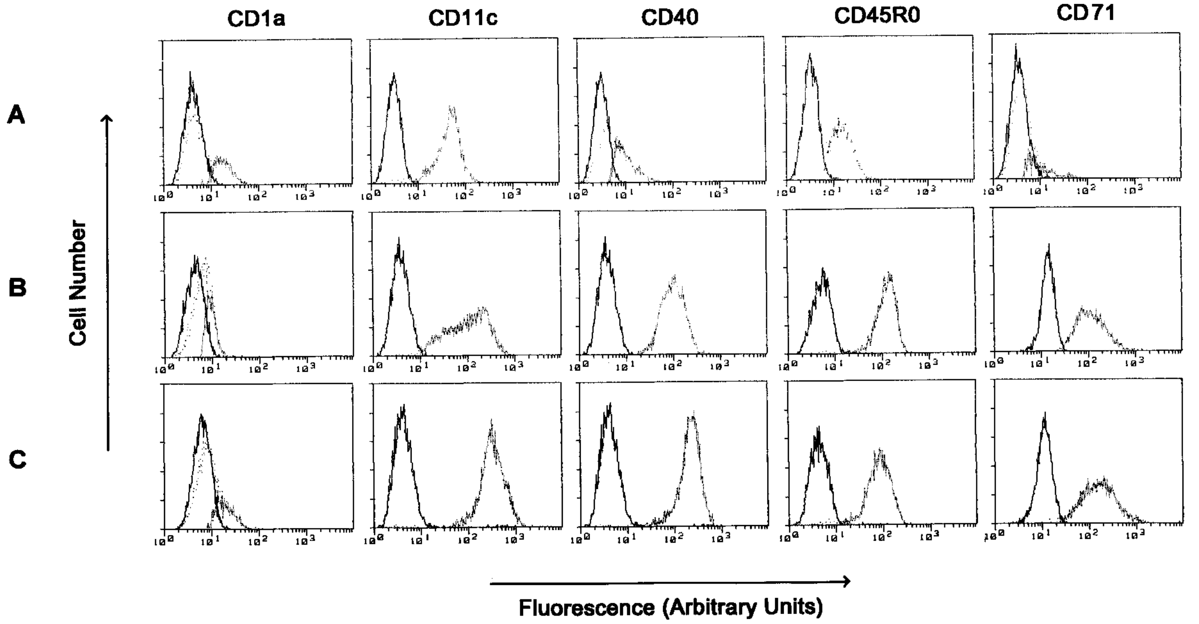

FIGURE 3 Expression of markers delineating subsets of DC, mature stages of DC differentiation, and cell activation. All specifications are as given in the caption to Figure 2.

for their mature stage—even more so, when bearing in mind that isolated blood DC, when cultured, reveal a CD11c$^+$CD45R0$^+$ phenotype. Approaching almost 100% of cultured cells, the MoDC generated in this present study were also CD11c$^+$CD45R0$^+$ double-positive (Figures 3C and 6). One further antigen clearly expressed by DC, but only weakly by monocytes, is the B-cell marker CD40 (e.g., Zhou and Tedder, 1995 and 1996). Here, almost 100% of MoDC were CD40$^+$ (Figures 3C and 6). Further, Andreesen et al. (1984) showed that CD71, a receptor for complexes of Fe/transferritin, is expressed depending on the degree of myelomonocytic differentiation. We have therefore chosen this marker as additional proof for a mature stage of the MoDC. As opposed to only marginal expression of CD71 by low numbers of fresh monocytes, close to 100% of the MoDC, and veiled cells were strongly positive for this receptor (Figures 3 and 6). The total status of antigens chosen as markers of DC subsets, as well as the degree of maturity and activation of MoDC was CD1a$^{-/dim}$, CD11c$^+$, CD40$^+$, CD45R0$^+$, and CD71$^+$.

### Antigen-presenting and Costimulatory Molecules

Gene products encoded by the MHC are prerequisite for the effective presentation of processed antigen. Brooks and Moore (1988) showed that HLA-DR, -DP, and -DQ are strongly expressed by blood DC, whereas monocytes are only weakly positive for class II products of the DP and DQ subloci. We here confirmed this finding for freshly isolated monocytes (Figure 4A), and cells cultured with GM-CSF, IL-4, and IFN-$\gamma$ exhibited a class-II phenotype similar to that observed with blood DC (Figure 4C). Also, although culture may lead to the expression of higher levels of HLA-DP and -DQ (Brooks and Moore, 1988, and Figure 4B), the percentages of monocytes positive for these gene products were evidently lower than those detected with the MoDC, of which virtually all cells coexpressed HLA-DR, -DP, and -DQ (Figure 6). Importantly, veiled cells suspended in culture expressed even higher amounts of HLA-DR and -DP, that is, a peak of strong DR expression on the far right of the histogram, and a shoulder of DP expression at $\Delta$MFI ~700 (Figure 4C). Similarly, the expression of MHC class I (HLA-A, -B, -C) strongly

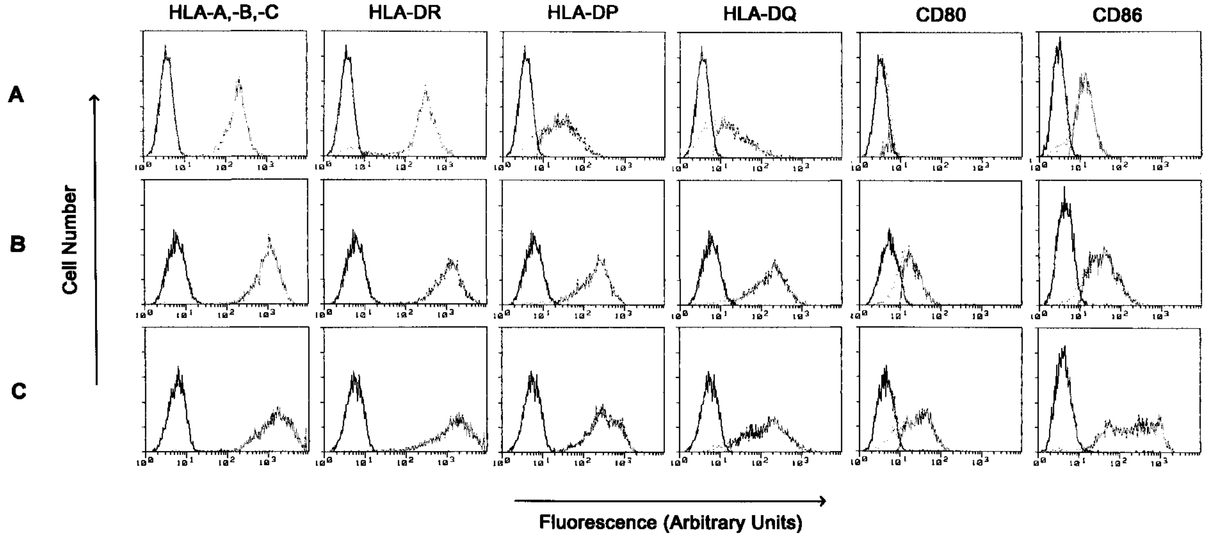

FIGURE 4 Flow-cytometric detection of MHC class I and class II as well as of costimulatory molecules. In a portion of GM-CSF/IL-4/IFN-$\gamma$ cells (row C), MHC class I and HLA-DR expression exceeded the logarithmic ΔMFI scale (i.e., >10000). These cells form seperate peaks that accumulate on the far right of the histograms. All specifications are as given for Fig. 2.

increased with the time of culture (Figure 4). Since it is known now that class I molecules not only serve to present endogenous products but also may participate in the presentation of exogenous antigens by M$\varphi$ and DC populations (Rock et al., 1993), an increase in class I obviously may augment the APC's antigen-presenting capacity. Moreover, paralleling the observations for class II, veiled cells revealed extremely upregulated class I expression, as reflected on the far right of the ΔMFI log scale (Figure 4C). Therefore, in strongly expressing MHC products, such cells qualify as potent APC. This is further underscored by the upregulation of costimulatory molecules B7.1 (CD80) and B7.2 (CD86) on MoDC, as compared to their starting population, or to cells cultured without cytokines (Figure 4). While being absent from immature DC (McLellan et al., 1995), the maturation of DC implies upregulation of CD86 as primary and CD80 as secondary costimulatory molecules to interact with their T-cell ligands CD28 or CTLA-4. Regular T-cell activation depends on these interactions, for their absence leads to anergy or apoptosis of the T cells involved (McLellan et al., 1995; Thompson, 1995). The breadth of the peak for CD86 indicates that, at this point of time, MoDC at least reach the B7.2-dependent stage of maturity (Figure 4C). As will be shown in what follows, such cells are potent stimulators of an allogeneic mixed-leukocyte reaction, whereas it is characteristic for immature DC that they are quiescent. Taken together, the MHC/costimulator phenotype of MoDC was MHC class $I^{++/+++}$, HLA-$DR^{++/+++}$, HLA-$DP^{+}$, HLA-$DQ^{+}$, $CD80^{dim}$ (but stronger than on M$\varphi$), and $CD86^{+/++}$.

### Adhesion Molecules

To conclude phenotypic characterization, we selected adhesion molecules, which, similar to B7.1 and B7.2, are essential for the successful stimulation of antigen-specific T cells by APC. Consistently, the Ig superfamily adhesion molecules CD11a ($\alpha_L$-chain) and CD11b ($\alpha_M$-chain), the $\beta$-chain CD18, as well as intercellular adhesion molecules (ICAM) 1 (CD54) and 3 (CD50) were all upregulated and expressed by ~90-100% of all MoDC Figures 5C and 6). As to CD11b, this molecule not only is a receptor for C3bi. For whereas CD11a and CD18 constitute the leukocyte function-associated molecule 1 (LFA-1), CD11b and CD18 can similarly associate as Mac-1. Both LFA-1 and Mac-1 may compete for interaction with ICAM-1 (Lub et al., 1996). We therefore will not speculate at the present time which function(s) of

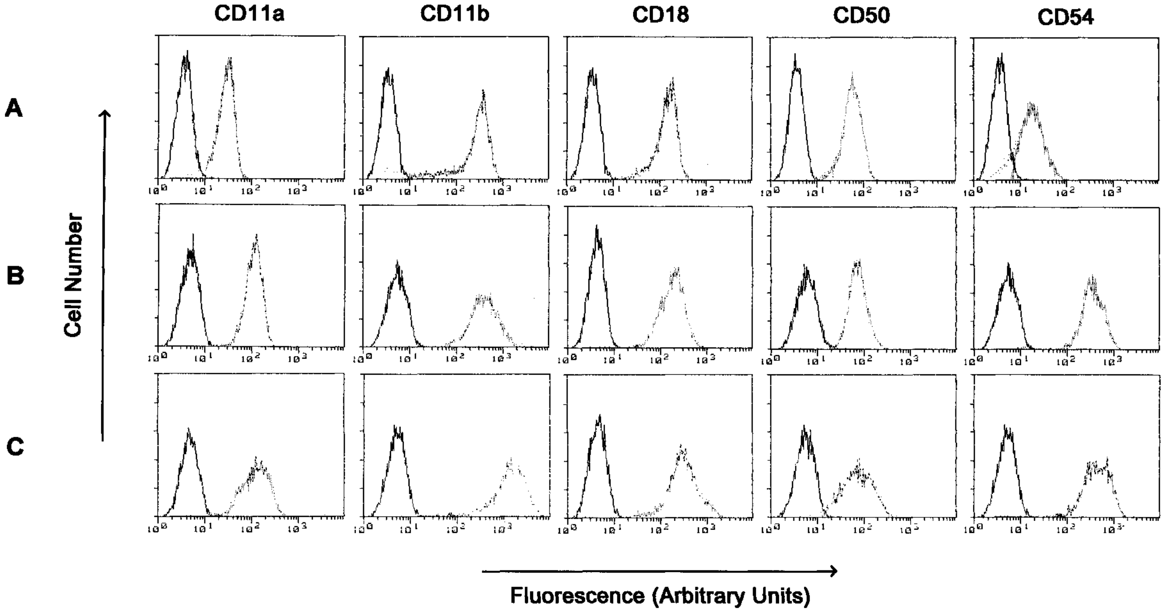

FIGURE 5 Cellular expression of selected adhesion molecules. For specifications, see above.

CD11b may be utilized by DC. However, it has been shown that $\alpha_M$-chains are expressed by subpopulations of DC (Franklin et al., 1986; Robinson et al., 1986), and not exclusively by M$\varphi$. Interestingly, ICAM-1 appears to be involved in DC migration and late DC-dependent T-cell stimulation (Starling et al., 1995). Therefore, this molecule may play a special role for mature DC, which would complement the previous picture. Indeed, ICAM-1 was found expressed by MoDC, and its broadened peak indicates different membrane densities of CD54 in the less mature DC and the more mature veiled cells. The pattern of selected adhesion molecules on MoDC was CD11a$^{+}$, CD11b$^{++}$, CD18$^{+}$, CD50$^{+}$, and CD54$^{+}$.

### The Functional Capacity of MoDC

To test the APC's potency to induce allospecific stimulation, we ran one-way mixed leukocyte cultures (MLC). Of the MoDC, both their adherent DC and suspended veiled-cell subsets were transferred into microwells and cocultured with pools of T lymphocytes. We observed the clustering of MoDC and T cells, as is typical of mature lymphoid DC (Austyn, 1987). MoDC induced a very vigorous proliferation of allogeneic T cells, which was not the case with fresh monocytes as stimulator cells (Figure 7). These data complement those presented in our previous study (Xu et al., 1995). Also, we recently were able to show that MoDC generated by action of GM-CSF, IL-4, and IFN-$\gamma$ are as well able to stimulate autologous antigen-specific T-cell proliferation (Soruri et al., 1998).

### DISCUSSION

The present study shows that DC and veiled cells can be generated from blood monocytes in the presence of GM-CSF, IL-4, and IFN-$\gamma$. Phenotypically, such cells are CD1a$^{-/dim}$, CD11a$^{+}$, CD11b$^{++}$, CD11c$^{+}$, CD14$^{dim/-}$, CD16a$^{-/dim}$, CD18$^{+}$, CD32$^{dim/-}$, CD33$^{+}$, CD40$^{+}$, CD45R0$^{+}$, CD50$^{+}$, CD54$^{+}$, CD64$^{-/dim}$, CD68$^{+}$, CD71$^{+}$, CD80$^{dim}$, CD86$^{+/++}$, MHC class I$^{++/+++}$, HLA-DR$^{++/+++}$, HLA-DP$^{+}$, and HLA-DQ$^{+}$. Specifically, the nonadherent cells exhibited morphologies that strongly resembled veiled-cell types that are in transit to, or encountered within, lymphoid tissues (examples are given in

Knight et al., 1986; Barfoot et al., 1989; and Pavli et al., 1993).

It is consistent with previous results that these MoDC—and, above all, the veiled cells—morphologically, phenotypically, and functionally represent a mature stage of differentiation. Irrespective of the cell lineage, CD45R0 is only expressed by activated or memory-type immunocytes. O'Doherty et al. (1993) were the first to show maturation of isolated blood DC into CD45R0$^+$ cells. They concluded that the highly stimulatory CD11c$^+$CD45R0$^+$ double-positive blood DC subset represents antigen-primed mature DC, which are possibly *en route* to lymphoid organs, while the less active CD11c$^-$CD45R0$^-$ subset apparently comprises immature blood DC (O'Doherty et al., 1994). Similarly, in addition to demonstrating that DC generated by action of GM-CSF, IL-4, and IFN-$\gamma$ are potent stimulators of allogeneic mixed leukocyte

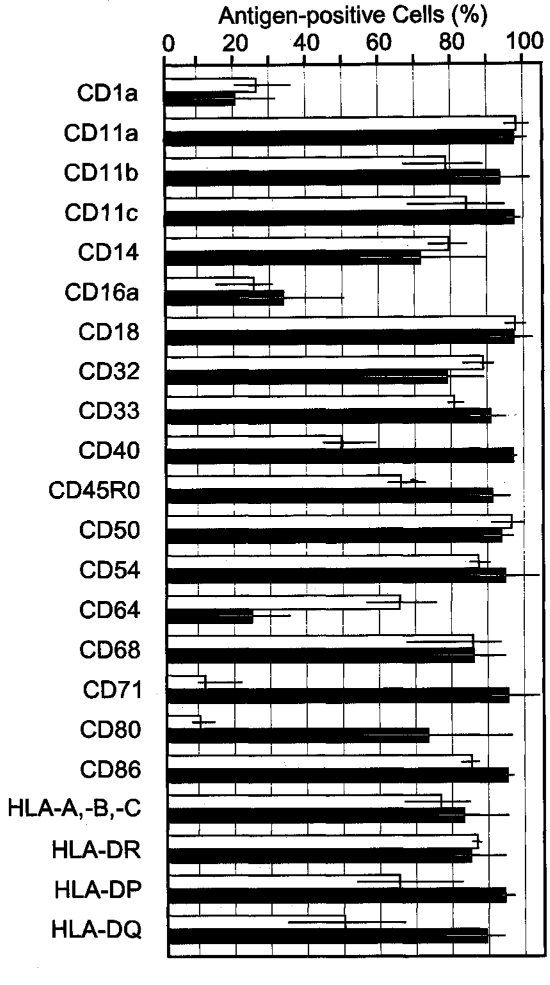

FIGURE 6 Percentages of antigen-positive cells. Data of fresh monocytes (empty bars) and cells cultured in the presence of GM-CSF/IL-4/IFN-$\gamma$ (solid bars) are given as means, with error bars indicating maxima and minima among the donors ($n$ = 4 to 6).

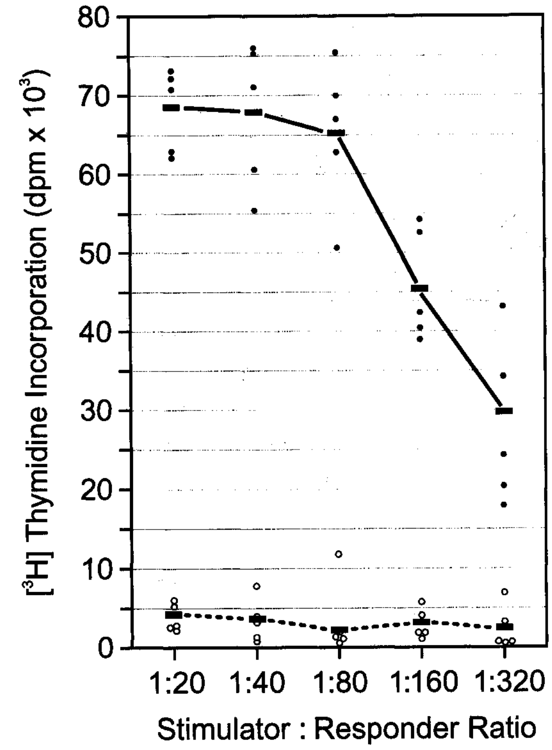

FIGURE 7 MoDC are potent stimulators of an allogeneic mixed-leukocyte reaction. Graded numbers (6.25 × $10^2$ to 1 × $10^4$) of fresh monocytes or MoDC generated by action of GM-CSF/IL-4/IFN-$\gamma$ were coincubated with 2 × $10^5$ allogeneic T cells. As opposed to monocytes (broken line, means and open symbols), the MoDC (solid line, means and closed symbols) were potent stimulator cells ($n$ = 5). T-cell controls revealed baseline incorporation of [$^3$H] thymidine (315 dpm). APC controls did not proliferate in any of the experiments. Consistent to all experiments, M$\varphi$ only stimulated marginally at a stimulator:responder ratio of 1:160 (529 dpm), and ceased to provoke T-cell proliferation at 1:320 (231 dpm).

reactions (Xu et al., 1995), we now show that such cells coexpress CD45R0 and CD11c. Another feature identifying mature DC is their CD14$^{dim}$CD33$^{bright}$ phenotype, as opposed to functionally immature CD14$^{dim}$CD33$^{dim}$ DC (Thomas et al., 1993; Thomas and Lipsky, 1994). Likewise, DC derived from monocytes under GM-CSF, IL-4 and IFN-$\gamma$ were CD14$^{dim/-}$CD33$^{+}$.

Zhou and Tedder (1995) showed that CD14$^{+}$ monocytes, when cultured with GM-CSF, IL-4, and TNF-$\alpha$, develop into CD83$^{+}$ DC. CD83 belongs to the immunoglobulin superfamily and is distinctive for mature blood DC. Of interest, in the absence of proliferation, the yield of these MoDC was similar to the number of monocytes cultured (Zhou and Tedder, 1996), which corroborates previous results (Peters et al., 1991), and is in line with the fact that bone-marrow precursors must pass a transient monocyte stage before acquiring DC characteristics (Gieseler et al., 1991). It was also observed that prolonged culture of MoDC leads to their transition into large, round M$\varphi$-like cells (Zhou and Tedder, 1996). This matches the finding that highly stimulatory DC, as derived from human monocytes in the presence of a placental medium supplement, can be triggered to develop into M$\varphi$ by action of M-CSF, whereby accessory activity is completely abrogated from such cultures (Gieseler, 1987). A serial monocyte $\rightarrow$ DC $\rightarrow$ M$\varphi$ transition could as well be achieved by the addition of serum to human or rat DC derived from myeloid precursors (Gieseler, 1987; Najar et al., 1990; Gieseler et al., 1991; Peters et al., 1991).

As to their course of differentiation, DC generated in the presence of GM-CSF, IL-4, and TNF-$\alpha$ first downregulate CD14, then become CD1a$^{+}$, again downregulate CD1a, and eventually express CD83. At this stage, the MoDC additionally acquire CD45R0 (Zhou and Tedder, 1996). Hence, cells differentiated with this cytokine mix reveal a transient CD1a$^{+}$ Langerhans cell phenotype and further proceed to develop into a mature interdigitating DC type.

Similarly, a mature DC phenotype, which may delineate cells in transit to or present within lymphoid organs, can also be induced with a cytokine cocktail employing GM-CSF, IL-4, and IFN-$\gamma$. Whether these cells express CD83 is currently investigated (CD83-specific mAb was kindly provided by F. Tedder, Duke University Medical Center, Durham, NC). First preliminary results indicate that they start to acquire CD83 on the sixth day of culture.

Do these MoDC relate to Langerhans cells? *In vivo*, skin DC express Mac-1 as well as Fc receptors (Steinman, 1991). Functionally, Mac-1 competes with LFA-1 to interact with ICAM-1 (Lub et al., 1996), which obviously plays a regulatory role in cell cooperation, and the Fc receptors may actively participate in the phagocytosis of antigens—a function clearly demonstrated for Langerhans cells (Reis e Sousa et al., 1993). These markers were likewise detected on the MoDC. That FcR were found downregulated indicates the MoDC's progression from immature cells that take up antigen to matured cells that may present processed antigenic material. This notion is not only supported by the parallel upregulation of MHC and costimulatory molecules, but also by the observation that such cells induce antigen-specific T-cell responses (Soruri et al., 1998). Another indication may be their CD1a$^{-/dim}$ status. Yet, there is a further interpretation that results from the microenvironment mimicked by Th1 and Th2 cytokines IFN-$\gamma$ and IL-4. Langerhanslike cells are not only present in skin, but also throughout mucosa-associated lymphoid tissue. Interestingly, enteric immune compartment provides a unique environment where the low-density subsets of both $\alpha/\beta$-TCR$^{+}$ and $\gamma/\delta$-TCR$^{+}$ intraepithelial lymphocytes spontaneously produce the Th1 mediator IFN-$\gamma$ (Yamamoto et al., 1993), whereas DC from Peyer's patches predominantly induce the production of Th2 cytokines such as IL-4 (Everson et al., 1996). We therefore suggest that monocyte-derived cells obtained by action of GM-CSF, IL-4, and IFN-$\gamma$ may resemble mucosal enteric DC. The retention of low amounts of CD32 Fc$\gamma$R II) on these cells, as opposed to human colonic DC *in situ* (Pavli et al., 1993), may well be due to the low concentration of cytokines employed. We currently address this issue in our experiments.

Most interestingly, and in contrast to epidermal Langerhans cells, DC freshly isolated from the colonic lamina propria are potent MLR stimulators

(Pavli et al., 1993). This feature identifies mucosal Langerhanslike cells as mature DC and is in line with their CD1a-negative phenotype (Graeme-Cook et al., 1993). It therefore is suggested that the mucosal-based lymphoid tissue functions as a genuine lymphoid organ that directly borders to the outer world.

That microenvironmental conditions imprint on the local cells' character is vividly demonstrated by experiments employing GM-CSF. This factor not only supports the differentiation of DC from monocytic precursors; it also effects the functional maturation of immature DC. Accordingly, GM-CSF induces isolated skin Langerhans cells to stimulate an immune response (Witmer-Pack et al., 1987), whereas, in contrast to this, liver-derived immature DC are induced to mature into tolerogenizing DC (Rastellini et al., 1995). Therefore, the functional repertory initiated by GM-CSF (and, probably, other factors) on terminal DC maturation obviously depends on the microenvironmental conditions shaping their immature precursors.

Correspondingly, to provide artificial microenvironments *in vitro* may allow to mimick conditions encountered in discrete tissues and has the potential to generate site-specific (and functionally diverse) DC subsets. Should the MoDC described in this study approximate to mucosal-type DC, they may serve as a starting point from which to proceed toward an optimized culture protocol.

Future prospects for such cells are evident. Some involve the establishment of *in-vitro* systems to mimick the tolerogenizing properties of the oral mucosa, which might be employed in the treatment of autoaggression. Others may allow a more specific investigation or treatment of mucosal-based immunopathologic disorders, such as the chronic inflammatory bowel diseases.

### MATERIALS AND METHODS

### Isolation and Culture of Monocytes

Buffy coats from healthy donors were kindly provided by the Blood Bank of the University Clinics and suspended 1:1 in phosphate-buffered saline (PBS) without $Ca^{2+}$ and $Mg^{2+}$ (Flow, Meckenheim, FRG). The suspensions were centrifuged through Lymphoprep ($\rho$ = 1.077 g/ml; Nycomed, Oslo) at 800 g for 20 min at RT. Interphases containing peripheral blood mononuclear cells (PBMNL) were collected and washed several times with cold PBS to remove platelets. PBMNL were then suspended in RPMI 1640 (Biochrom, Berlin), supplemented with 2 mM L-glutamine (Biochrom), and 100 U/ml penicillin plus 100 U/ml streptomycin (Pen/Strep; Flow) at 5% normal human serum (NHS).

Five-milliliter volumes of $2.5 \times 10^7$ cells were placed in hydrophobic 50-mm Teflon Petriperm dishes (Bachhofer, Reutlingen, FRG), precoated with human plasma that was obtained from whole blood preparations. The PBMNL were incubated for adherence under occasional agitation. After 40-45 min, nonadherent cells were rinsed off, and successful enrichment of monocytes (> 95%) was verified microscopically. On average, 10% of the initial cell suspension was adherent under these conditions, that is, $\sim 2.5 \times 10^6$ monocytes remained per culture dish. Monocytes were then added fresh RPMI 1640 with Pen/Strep and 5% NHS, containing GM-CSF (100 IU/ml), IL-4 (100 IU/ml), and IFN-$\gamma$ (50 IU/ml) (Genzyme, Cambridge, UK). The cells were incubated for 6 days to differentiate into MoDC. Monocytes were also cultured without cytokines.

For phenotypical and functional analyses, freshly isolated monocytes or adherent cells in culture were detached by 1-hr incubation with cold PBS/0.01% EDTA. Suspended cells were collected as well. After several washes at 350 g in the cold, cells were adjusted to concentrations detailed in the respective method.

The viability of enriched monocytes or cultured cells was determined with propidium iodide at 5 mg/ml. The substance intercalates with cellular DNA when infiltrating dead or damaged cells. Incubated cells were immediately analyzed flow-cytometrically for red fluorescence ($\lambda$ = 570 nm) of nuclei. General viability was > 95%; occasional cultures exhibiting lower viability rates were excluded from the experiments.

### Isolation, Pooling, and Storage of T Cells

Sheep blood was diluted 1:1 with PBS (RT) and washed several times for 12 min at 1630 g and RT. The erythrocyte pellet was then suspended in RPMI 1640 and stored overnight at 4°C. Red cells were used for CD2-dependent rosetting of T cells. To enhance erythrocyte-T-cell interaction, nonadherent PBMNL were washed and suspended at 2 × $10^7$/ml in 1% (w/v) polyethylene glycol dissolved in RPMI 1640/5% NHS. The suspension was then added 10% (v/v) erythrocyte solution. Cells were carefully mixed, sedimented for 10 min at 350 g, and T-cell rosetting was carried out for 1 hr in the dark at 4°C. Successively, pellets were carefully resuspended with 12 ml PBS (RT) to preserve rosettes, layered onto Lymphoprep gradients, and centrifuged for 18 min at 800 g. While discarding the supernatant and nonrosetted interphase cells, pelleted T-cell — erythrocyte rosettes were resuspended in PBS and washed three times. Rosettes were then suspended in 5-10 ml erythrocyte lysis buffer (Gieseler et al., 1991) and incubated for 15-30 min at 37°C. Thereafter, T lymphocytes were obtained by several washes to remove red-cell debris. T-cell's viability was > 95% in all of the cases, as determined by trypan blue exclusion.

To employ T cells as allogeneic MLC responder cells, we prepared T-cell pools from four to six donors. Aliquots of 2.5 × $10^7$ T cells per 450 μl FCS (PAA Biologics, Marburg, FRG) were then portioned into precooled cryotubes (Nunc, Wiesbaden, FRG). Immediately after adding 50 μl DMSO each, the tubes were frozen to –70°C at 1-2°C/min. Pools were used no later than 30 days after storage. Viability of thawed T-cell pools, as determined by trypan blue exclusion, was ~90%.

### Scanning Electron Microscopy

To preserve general cell morphology and veil expression, we avoided time-consuming preparatory steps and physical alterations. Round 8-mm coverslips were precoated with 0.01% poly-L-lysine in deionized water and covered with droplets of 2% glutaraldehyde in 0.1 M cacodylate buffer. Nonadherent monocyte-derived veiled cells were then carefully collected from cultures with a Pasteur pipette, and concentrated in the pipette tip by 1-g sedimentation. Small volumes of cell suspension were applied on top of the glutaraldehyde droplets. Thus, the cells were carefully fixed while sedimenting, and were successively immobilized on the poly-L-lysine-coated glass surface. The fixative was removed after 2 hr. After dehydration in graded ethanol, samples were dried in a critical point dryer (Polaron, Watford, UK), mounted on stubs, and coated with gold/palladium in a cool sputter coater (Fisons Instruments, Uckfield, UK). Micrographs were taken with a DSM 960 scanning electron microscope (Zeiss, Oberkochen, FRG).

### Flow Cytometry

All steps were carried out at 4°C. Monoclonal antibodies (mAb) used are given in Table I. Adherent and suspended cells were collected and transferred to 96-well round-bottom microtiter plates (Nunc) at 1 × $10^5$ cells/100 μl. To keep all cells suspended, wells were precoated for 30 min with 200 μl blocking buffer (10% heat-inactivated rabbit serum and 0.1% $NaN_3$ in PBS). Cells were sedimented and incubated for 30-45 min in blocking buffer.

For direct staining, each well received 5 μl of phycoerythrin- (PE) conjugated anti-CD64 (1:10 in washing buffer, i.e., 1% BSA and 0.1% $NaN_3$ in PBS). For direct double staining (CD1a × fluorescein isothiocyanate [FITC], CD3 × PE, CD19 × PE, CD71 × FITC), the cells were resuspended in 50 μl washing buffer, and were added 5 μl of PE conjugate plus 5 μl of FITC conjugate. The plates were incubated for 45 min, and washed thereafter. For storage of up to 2 days, stained cells were fixed with 200 μl/well of 1% formaldehyde in PBS, sealed, and stored in the dark.

All other antigens (cf. Table I) were stained indirectly, by either adding 50 μl/well of first mAb

TABLE I Mouse Anti-Human mAb Used for the Flow-Cytometric Characterization of Monocyte-Derived DC

| CD | Other names[a] | Clone | Isotype | Source |
|---|---|---|---|---|
| CD1a | — | OKT6 (SK9) | IgG1 | Ortho |
| CD11a | LFA-1α | 25.3.1 | IgG1 | Immunotech |
| CD11b | CR3, Mac-1α | BEAR1 | IgG1 | Dianova |
| CD11c | p150/90-α | BU-15 | IgG1 | Immunotech |
| CD14 | — | Hb44 (63D3) | IgG1 | ATCC |
| CD16a | FcγR IIIA | CLB-149 | IgG2a | Janssen |
| CD18 | LFA-1β | BL5 | IgG1 | Dianova |
| CD32 | FcγR II | 2E1 | IgG2a | Dianova |
| CD33 | — | WM54 | IgG1 | Serotec |
| CD40 | — | EA-5 | IgG1 | Serotec |
| CD45R0 | PTP isoform | UCHL1 | IgG2a | Serotec |
| CD50 | ICAM-3 | KS128 | IgG1 | Dako |
| CD54 | ICAM-1 | 84H10 | IgG1 | Immunotech |
| CD64 | FcγR I | 10.1 | IgG1 | PharMingen |
| CD68 | — | EBM11 | IgG1 | Dako |
| CD71 | TfR | YDJ1.2.2 | IgG1 | Immunotech |
| CD80 | B7-1 | MAB104 | IgG1 | Dianova |
| CD86 | B7-2 | BU-63 | IgG1 | Serotec |
| — | HLA-A,B,C | Hb95 (W6/32) | IgG2a | ATCC |
| — | HLA-DR | B8.12.2 | IgG2b | Immunotech |
| — | HLA-DP | B7/21 | IgG1 | Becton-Dickinson |
| — | HLA-DQ | SK10 | IgG1 | Becton-Dickinson |

[a]B7-1, costimulatory ligand for CD28 and CTLA-4; CR3, complement receptor type 3; FcγR I, II, and IIIA, Fc receptors for IgG; gp45, member of the integrin superfamily; HLA-A, -B, -C, MHC class I antigens; HLA-DR, -DP, -DQ, MHC class II antigens; ICAM-1 and -3, intercellular adhesion molecules 1 and 3; LFA-1α and -1β, leukocyte function-associated antigen $\alpha_L$- and β-chain; Mac-1α, integrin $\alpha_M$-chain; p150/90-α, integrin $\alpha_X$-chain; PTP, protein tyrosin phosphatase (a.k.a. leukocyte common antigen, T200); TfR, transferrin receptor.

(1:50 in washing buffer) or 100 μl/well of undiluted hybridoma supernatant per well. After an incubation for 45 min in the dark, the plates were centrifuged, depleted of antibody solution, washed twice, and supplied with 50 μl/well of 1:50 second rabbit anti-mouse IgG $F(ab')_2 \times$ PE (Dianova, Hamburg). The plates were again incubated for 45 min in the dark, and final processing was as described earlier.

Negative controls for direct staining employed irrelevant mouse IgG1 × FITC or murine IgG2a × PE (Dianova). Nonsense mouse anti-human IgG TIB-8 hybridoma supernatant (ATCC, Rockville, MD) was used as a negative control for indirect staining.

Cellular antigen expression was measured with the FACStar$^{PLUS}$ Type IV (Becton-Dickinson, Erembodegem-Aalst, Belgium). FITC-conjugated mAb were detected at $\lambda$ = 530 nm, whereas PE conjugates were measured at $\lambda$ = 570 nm. Cell fragments were excluded by adjusting the particle-size threshold. Occasional autofluorescence was subtracted from the respective determinations. Data were then evaluated with the C30 calculation program (Becton-Dickinson), resulting in percentages of antigen-positive cells per specimen, as well as mean fluorescence intensities (ΔMFI), which were determined using the subtract-graph option according to Werfel et al., (1991).

$$\Delta MFI = MFI_{SAMPLE} - MFI_{CONTROL} \quad (1)$$

Controls for T cells (anti-CD3; UCHT1, IgG1; Immunotech), B cells (anti-CD19, J4.119, IgG1; Immunotech), NK cells (anti-CD56, B-A19, IgG1; Serotec), and granulocytes (anti-CD66, 80H3, IgG1;

Immunotech) were negative in all of the cases (not demonstrated).

### Allogeneic One-Way MLC

MLC tests were run in 96-well flat-bottomed microtiter plates (Nunc). Cells detached from Teflon dishes were adjusted to $2 \times 10^5$/ml in RPMI 1640 plus 10% FCS. Stimulator cells were then plated at $6.25 \times 10^2$ to $2 \times 10^4$ per well, irradiated at 1500 rad, and coincubated with $2 \times 10^5$/well allogeneic responder T cells (pretested for reactivity). Negative controls omitted responders or stimulators. After 5 days, each culture well was added 1.0 $\mu$Ci (37 kBq) of [$^3$H]thymidine (Amersham, Braunschweig, FRG), and the tests were stopped 24 hr later. Cells were then harvested using an automated Inotech cell harvester (Dunn, Asbach, FRG), and thymidine incorporation was determined with a Matrix 96 Direct $\beta$ Counter (Hewlett-Packard, Meriden, CT). Results are expressed as [dpm].

### *Acknowledgements*

The authors are grateful to R. Kuhn for expert technical assistance, to F. Djalali Bazzaz for participating in the preparation of SEM micrographs, and to all our colleagues, especially H.-P. Spengler, for stimulating discussions and critical comments. Special thanks are to S. Lenzner for preparing Figures. 6 and 7. We also thank E. Pralle and G. Kasten for their helpful support. This study was supported by Research Award from the Crohn's and Colitis Foundation of Germany (DCCV), sponsored by Astra GmbH (Wedel, FRG), to R.G., and by grants SFB 500-C4 and Pe 192/6-2 from the Deutsche Forschungsgemeinschaft.

### *References*

Andreesen R., Osterholz J., Bodemann H., Bross K.J., Costabel U, and Lohr G.W. (1984). Expression of transferrin receptors and intracellular ferritin during terminal differentiation of human monocytes. Blood **49**: 195-202.

Austyn J.M. (1987). Lymphoid dendritic cells. Immunology **62**: 161-170.

Ardavin C., Wu L., Li C.-L., and Shortman K. (1993). Thymic dendritic cells and T cells develop simultaneously in the thymus from a common precursor population. Nature **362**: 761-763.

Barfoot R., Denham S., Gyure L.A., Hall J.G., Hobbs S.M., Jackson L.E., and Robertson D. (1989). Some properties of dendritic macrophages from peripheral lymph. Immunology **68**: 233-239.

Beelen R.H.J., Steenbergen J.J.E., van Vugt E., Betjes M.G.H., Havenith C.E.G., and Kamperdijk E.W.A. (1993). Dendritic cells isolated from rat and human non-lymphoid tissue are very potent accessory cells. Adv. Exp. Med. Biol. **329**: 123-127.

Brooks C.F., and Moore M. (1988). Differential MHC class II expression on human peripheral blood monocytes and dendritic cells. Immunology **63**: 303-311.

Chen-Woan M., Delaney C.P., Fournier V., Wakizaka Y., Murase N., Fung J., Starzl T.E., and Demetris A.J. (1996). In vitro characterization of rat bone marrow-derived dendritic cells and their precursors. J. Leukoc. Biol. **59**: 196-207.

Everson M.P., McDuffie D.S., Lemak D.G., Koopman W.J., McGhee J.R., and Beagley K.W. (1996). Dendritic cells from different tissues induce production of different T cell cytokine profiles. J. Leukoc. Biol. **59**: 494-498.

Franklin W.A., Mason D.Y., Pulford K., Falini B., Bliss E., Gatter K.C., Stein H., Clarke L.C., and McGee J.O. (1986). Immunohistological analysis of human mononuclear phagocytes and dendritic cells by using monoclonal antibodies. Lab. Invest. **54**: 322-335.

Gieseler R.K.H. (1987). In vitro-Induktion von akzessorischen Immunzellen bei Maus, Ratte und Mensch. Ph.D. diss., Georg-August University, Göttingen.

Gieseler R., Heise D., Heinemann D.E.H., and Peters J.H. (1998). Mucosal dendritic cells and $CD14^{low}CD16a^+$ inflammatory macrophages generated in vitro: implications for ulcerative colitis and AIDS. Clin Exp. Immunol. (in press).

Gieseler R.K.H., Röber R.-A., Kuhn R., Weber K., Osborn M., and Peters J.H. (1991). Dendritic accessory cells derived from rat bone marrow precursors under chemically defined conditions in vitro belong to the myeloid lineage. Eur. J. Cell Biol. **54**: 171-181.

Graeme-Cook F., Bhan A.K., and Harris N.L. (1993). Immunohistochemical characterization of intraepithelial and subepithelial mononuclear cells of the upper airways. Am. J. Pathol. **143**: 1416-1422.

Inaba K., Inaba M., Deguchi M., Hagi K., Yasumizu R., Ikehara S., Muramatsu S., and Steinman R.M. (1993). Granulocytes, macrophages and dendritic cells arise from a common major histocompatibility complex class II-negative progenitor in mouse bone marrow. Proc. Natl. Acad. Sci. USA **90**: 3038-3042.

Kabel P.J., De Haan-Meulmann M., Voorbij H.A.M., Kleingeld M., Knol E.F., and Drexhage H.A. (1989). Accessory cells with the morphology and marker pattern of dendritic cells can be obtained from elutriator-purified blood monocyte fractions: an enhancing effect of metrizamide in this differentiation. Immunobiology **179**: 395-410.

Kiertscher S.M., and Roth M.D. (1996). Human $CD14^+$ leukocytes acquire the phenotype and function of antigen-presenting dendritic cells when cultured in GM-CSF and IL-4. J. Leukoc. Biol. **59**: 208-218.

Knight S.C., Farrant J., Bryant A., Edwards A.J., Burman S., Lever A., Clarke J., and Webster D.B. (1986). Nonaderent, low-density cells from human peripheral blood contain dendritic cells and monocytes, both with veiled morphology. Immunology **57**: 595-603.

Lub M., van Kooyk Y., and Figdor C.G. (1996). Competition between lymphocyte function-associated antigen 1 (CD11a/CD18) and Mac-1 (CD11b/CD18) for binding to intercellular adhesion molecule-1 (CD54). J. Leukoc. Biol. **59**: 648-655.

McLellan A.D., Starling G.C., Williams L.A., Hock B.D., and Hart D.N.J. (1995). Activation of human peripheral blood dendritic cells induces the CD86 costimulatory molecule. Eur. J. Immunol. **25**: 2064-2068.

Najar H.M., Bru-Capdeville A.C., Gieseler R.K.H., and Peters J.H. (1990). Differentiation of human monocytes into accessory cells at serum-free conditions. Eur. J. Cell Biol. **51**: 339-346.

O'Doherty U., Peng M., Gezelter S., Swiggard W.J., Betjes M., Bhardwaj N., and Steinman R.M. (1994). Human blood contains two subsets of dendritic cells, one immunologically mature and the other immature. Immunology **82**: 487-493.

O'Doherty U., Steinman R.M., Peng M., Cameron P.U., Gezelter S., Kopeloff I., Swiggard W.J., Pope M., and Bhardwaj N. (1993). Dendritic cells freshly isolated from human blood express CD4 and mature into typical immunostimulatory dendritic cells after culture in monocyte-conditioned medium. J. Exp. Med. **178**: 1067-1076.

O'Neill H.C. (1994). The lineage relationship of dendritic cells with other haematopoietic cells. Scand. J. Immunol. **39**: 513-516.

Pavli P., Hume D.A., van de Pol E., and Doe W.F. (1993). Dendritic cells, the major antigen-presenting cells of the human colonic mucosa. Immunology **78**: 132-141.

Peters J.H., Gieseler R., Thiele B., and Steinbach F. (1996). Dendritic cells: from ontogenetic orphans to myelomonocytic descendants. Immunol. Today **17**: 273-278.

Peters J.H., Ruhl S., and Friedrichs D. (1987). Veiled accessory cells deduced from monocytes. Immunobiology **176**: 154-166.

Peters J.H., Ruppert J., Gieseler R.K.H., Najar H.M., and Xu H. (1991). Differentiation of human monocytes into CD14 negative accessory cells: Do dendritic cells derive from the monocytic lineage? Pathobiology **59**: 122-126.

Porcelli S., Morita C.T., and Brenner M.B. (1992). CD1b restricts the response of human $CD4^-8^-$ T lymphocytes to a microbial antigen. Nature **360**: 593-597.

Rastellini C., Lu L., Ricordi C., Starzl T.E., Rao A.S. and Thomson A.W. (1995). Granulocyte/macrophage colony-stimulating factor-stimulated hepatic dendritic cell progenitors prolong pancreatic islet allograft survival. Transplantation **60**: 1366-1370.

Reid C.D., Stackpoole A., Meager A., and Tikerpae J. (1992). Interactions of tumor necrosis factor with granulocyte-macrophage colony-stimulating factor and other cytokines in the regulation of dendritic cell growth in vitro from early bipotent $CD34^+$ progenitors in human bone marrow. Immunology **149**: 2681-2688.

Reis e Sousa C., Stahl P., and Austyn J. (1993). Phagocytosis of antigens by Langerhans cells in vitro. J. Exp. Med. **178**: 509-519.

Robinson A.P., White T.M., and Mason D.W. (1986). Macrophage heterogeneity in the rat as delineated by two monoclonal antibodies MRC OX-41 and MRC OX-42, the latter recognizing complement receptor type 3. Immunology **57**: 239-247.

Rock K.L., Rothstein L., Gamble S., and Fleischacker C. (1993). Characterization of antigen-presenting cells that present exogenous antigens in association with class I MHC molecules. J. Immunol. **150**: 438-446.

Romani N., Gruner S., Brang D., Kämpgen E., Lenz A., Trockenbacher B., Kowalinka G., Fritsch P.O., Steinman R.M., and Schuler G. (1994). Proliferating dendritic cell progenitors in human blood. J. Exp. Med. **180**: 83-93.

Ruppert J., Schütt C., Ostermeier D., and Peters J.H. (1993). Down-regulation and release of CD14 on human monocytes by IL-4 depends on the presence of serum or GM-CSF. Adv. Exp. Med. Biol. **329**: 281-286.

Sallusto F., and Lanzavecchia A. (1994). Efficient presentation of soluble antigen by cultured human dendritic cells is maintained by granulocyte/macrophage colony-stimulating factor plus interleukin 4 and downregulated by tumor necrosis factor $\alpha$. J. Exp. Med. **179**: 1109-1118.

Santiago-Schwarz F., Belilos E., Diamond B. and Carsons S.E. (1992). TNF in combination with GM-CSF enhances the differentiation of neonatal cord blood stem cells into dendritic cells and macrophages. J. Leukoc. Biol. **52**: 274-281.

Soruri A., Fayyazi A., Gieseler R., Schlott T., Rünger T.M., Neumann C., and Peters J.H. (1998). Specific autologous anti-melanoma T cell response *in vitro* using monocyte-derived dendritic cells. Immunobiology **198:** 39–50.

Starling G.C., McLellan A.D., Egner W., Sorg R.V., Fawcett J., Simmons D.L., and Hart D.N.J. (1995). Intercellular adhesion molecule-3 is the predominant costimulatory ligand for leukocyte function antigen-1 on human blood dendritic cells. Eur. J. Immunol. **25**: 2528-2532.

Steinbach F., Krause B., and Thiele B. (1995). Monocyte-derived cells (MoDC) represent phenotype and functional activities of Langerhans cells/dendritic cells. Adv. Exp. Med. Biol. **378**: 151-153.

Steinman R.M. (1991). The dendritic cell system and its role in immunogenicity. Annu. Rev. Immunol. **9**: 271-296.

Steinman R.M., and Cohn Z. (1973). Identification of a novel cell type in peripheral lymphoid organs of mice. I. Morphology, quantitation and tissue distribution. J. Exp. Med. **137**: 1142-1162.

Thomas R., Davis L.S., and Lipsky P.E. (1993). Isolation and characterization of human peripheral blood dendritic cells. J. Immunol. **150**: 821-834.

Thomas R. and Lipsky P.E. (1994). Human peripheral blood dendritic cell subsets: isolation and characterization of precursor and mature antigen-presenting cells. J. Immunol. **153**: 4016-4072.

Thompson C.B. (1995). Distinct roles for the costimulatory ligands B7-1 and B7-2 in T helper cell differentiation? Cell **81**: 979-982.

Werfel T., Sonntag G., Weber M.H., and Götze O. (1991). Rapid increases in the membrane expression of neutral endopeptidase (CD10), aminopeptidase N (CD13), tyrosine phosphatase (CD45), and Fc$\gamma$RIII (CD16) upon stimulation of human peripheral leucocytes with human C5a. J. Immunol. **147**: 3909-3914.

Witmer-Pack M., Olivier W., Valinsky J., Schuler G., and Steinman R. (1987). Granulocyte/macrophage colony-stimulating factor is essential for the viability and function of cultured murine epidermal Langerhans cells. J. Exp. Med. **166**: 1484-1489.

Xu H., Krämer M., Spengler H.-P., and Peters J.H. (1995). Dendritic cells differentiated from human monocytes through a combination of IL-4, GM-CSF and IFN-$\gamma$ exhibit phenotype and function of blood dendritic cells. Adv. Exp. Med. Biol. **378**: 75-78.

Yamamoto M., Fujihashi K., Beagley K.W., McGhee J.R., and Kiyono H. (1993). Cytokine synthesis by intestinal intraepithelial lymphocytes. Both $\gamma/\delta$ T cell receptor-positive and $\alpha/\beta$ T cell receptor-positive T cells in the G1 phase of cell cycle produce IFN-$\gamma$ and IL-5. J. Immunol. **150**: 106-114.

Zhou L.-J., and Tedder T.F. (1995). Human blood dendritic cells selectively express CD83, a member of the immunoglobulin superfamily. J. Immunol. **154**: 3821-3835.

Zhou L.-J., and Tedder T.F. (1996). $CD14^+$ blood monocytes can differentiate into functionally mature $CD83^+$ dendritic cells. Proc. Natl. Acad. Sci. USA **93**: 2588-2592.

